# Characterization of the Antibacterial Activity and the Chemical Components of the Volatile Oil of the Leaves of *Rubus parvifolius* L.

**DOI:** 10.3390/molecules17077758

**Published:** 2012-06-25

**Authors:** Yongqing Cai, Xiaogang Hu, Mingchun Huang, Fengjun Sun, Bo Yang, Juying He, Xianfeng Wang, Peiyuan Xia, Jianhong Chen

**Affiliations:** Department of Pharmacy, Southwest Hospital, Third Military Medical University, Chongqing 400038, China; Email: cy0721@163.com (Y.C.); hxgcq1987@126.com (X.H.); xyjk11@sina.com (M.H.); cm20030409@yahoo.com.cn (F.S.); tmmuyb1@163.com (B.Y.); hejuying95@126.com (J.H.); wxf3111@126.com (X.W.)

**Keywords:** *Rubus parvifolius* L., volatile oil, 4-hydroxy-3-methoxystyrene, antibacterialactivity

## Abstract

*Rubus parvifolius* L. (Rp) is a medicinal herb that possesses antibacterial activity. In this study, we extracted the volatile oil from the leaves of Rp to assess its antibacterial activity and analyze its chemical composition. A uniform distribution design was used to optimize the extraction procedure, which yielded 0.36% (*w*/*w*) of light yellowish oil from the water extract of Rp leaves. We found that the extracted oil effectively inhibited the growth of a wide range of Gram positive and negative bacteria, including *Staphylococcus aureus*, *Staphylococcus epidermidis*, *Enterococcus faecalis*, *Escherichia coli*, *Pseudomonas aeruginosa*, *Acinetobacter baumanii*, *Bacillus cloacae*, and *Klebsiella pneumoniae*. We further analyzed the components contained in the hydro-distillated Rp volatile oil by gas chromatography-mass spectroscopy. Twenty nine compounds were identified, including 4-hydroxy-3-methoxystyrene (66%), 3,7,11,15-tetramethyl-2-hexadecen-1-ol (10%) and 4-*tert*-butylbenzoic acid (2%). Our results suggest that one or multiple constituents contained in Rp volatile oil may account for its antibacterial activity.

## 1. Introduction

*Rubus parvifolius* L. (Rp) is a deciduous thorny shrub widely distributed in East and South Asia, and used in herbal medicines for the treatment of many inflammatory and infectious diseases [1,2]. Previous studies demonstrated that the extracts of Rp were effective in shortening bleeding and coagulation time, and increasing tolerance to hypoxia [3,4,5]. The extracts also showed hepatoprotective effects and functioned as antioxidants [6,7,8,9,10]. The leaves of Rp can inhibit the growth of *E. coli* and *Pasteurella* [11]. In addition, Rp volatile oil is effective against other bacteria, including *Staphylococcus aureus*, *Staphylococcus epidermidis*, *Bacillus subtilis*, *Micrococcus luteus*, *White candidiasis* and *Enterococcus faecalis* [12,13]. However, the detailed chemical composition responsible for the biological activities of the volatile oil that is extracted from the leaves of Rp remains elusive.

To investigate the antibacterial activity of the volatile oil extracted from the leaves of Rp, in this study we used a uniform distribution design to optimize the volatile oil extraction method. The light-yellow oil obtained from Rp leaves was tested for its antibacterial activity. We further applied gas chromatography-mass spectrometry (GC-MS) to determine the contents of Rp volatile oil. Our study suggests that the Rp volatile oil and its components could be utilized as drug-leads for the development of novel anti-inflammatory and antibacterial agents.

## 2. Results and Discussion

### 2.1. Uniform Distribution Experimental Design of Extraction

**Figure 1:**
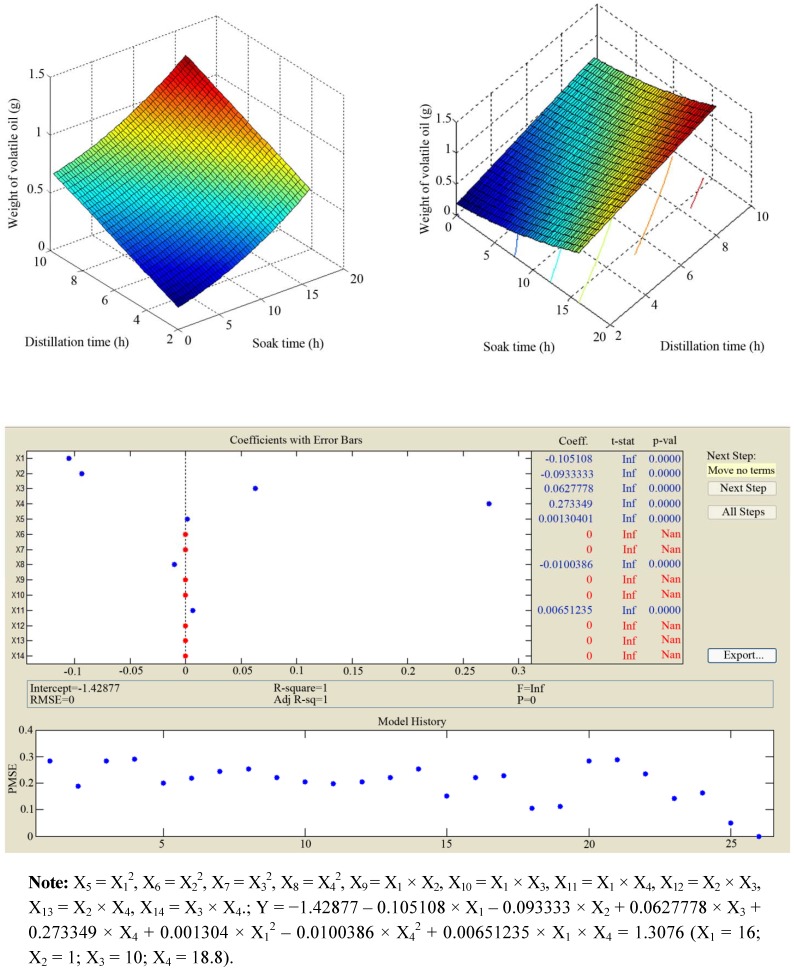
Results of the uniform distribution experimental design.

Previous studies found that the yield of volatile oil extracted from *Rubus parvifolius* L. was very low [11]. In our study, we applied a Clevenger apparatus to achieve a higher yield. NaCl (80 g/L) was also added during the extraction. A uniform distribution experimental design was used to optimize the extraction efficiency (Table 1 and Figure 1).

**Table 1:** U9 (3^4^) Uniform distribution of volatile oil extracted from *Rubus parvifolius* L. leaves.

No.	Soak time X_1_ (h)	Grinding degree X_2_ (mesh)	Distillation time X_3_ (h)	Ratio of solvent to leaves X_4_ ( *v*/*w*)	Weight of volatile oil (g)
1	0	< 20	5	20	0.15
2	2	20–40	9	16	0.85
3	4	> 40	4	12	0.29
4	6	< 20	8	8	0.16
5	8	20–40	3	22	0.21
6	10	> 40	7	18	0.65
7	12	< 20	2	14	0.39
8	14	20–40	6	10	0.28
9	16	> 40	10	24	0.85

Regression analysis indicated that independent variable tests were statistically significant (*p *< 0.05). Combination of errors value was 0 with adjustment R^2^ of 1, indicating that all the experimental points were predicted by the regression equation. Thus, the equation was accurate and effective. The optimised conditions were that the 20-mesh filtered leave powders were soaked with 18.8 times purified water for 16 h, and then boiled at 100 °C for 10 h. The optimal extraction yielded 0.36% (*w/w*) of light yellowish oil. Our results showed that the yield of Rp volatile oil was higher than that of previous studies [11,14].

### 2.2. Antibacterial Activity of Rp Volatile Oil

**Figure 2:**
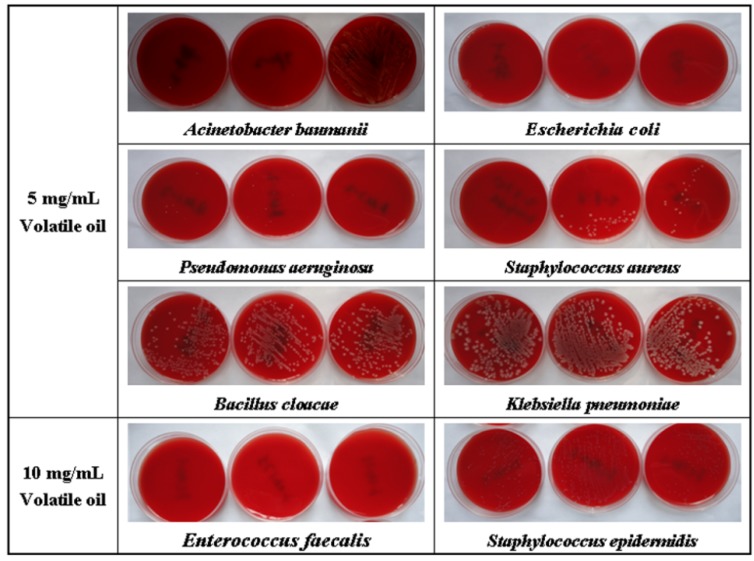
Minimal bactericidal concentration of Rp volatile oil.

Minimum inhibitory concentration (MIC) is commonly used to evaluate antibacterial activity. In our study, the MICs of Rp volatile oil on several bacterial strains were tested to determine their antibacterial potential. We found that the Rp volatile oil exhibited antibacterial activity (Table 2 and Figure 2).

**Table 2:** Antibacterial activity of volatile oilextracted from *Rubus parvifolius* L. leaves.

Bacterial species	Source	MIC (mg/mL)	MBC (mg/mL)
Gram-positive bacteria			
*Staphylococcus aureus*	CIS ^a^	5	> 5
*Staphylococcus aureus*	ATCC ^b^29213	5	10
*Staphylococcus epidermidis*	CIS	10	10
*Enterococcus faecalis*	CIS	10	10
Gram-negative bacteria			
*Escherichia coli*	CIS	5	5
*Escherichia coli*	ATCC 25922	5	5
Pseudomonas aeruginosa	CIS	> 2.5	5
Pseudomonas aeruginosa	ATCC 27853	2.5	5
*Acinetobacter baumanii*	CIS	> 2.5	5
*Bacillus cloacae*	CIS	5	10
*Klebsiella pneumoniae*	CIS	5	> 10

^a^ MIC: Minimal inhibitory concentration; ^b^MBC: Mimimal bactericidal concentration; ^c^ CIS: Clinically Isolated Strains, Department of Pharmacy, Southwestern Hospital (China); ^d^ ATCC: American Type Culture Collection (USA).

The MICs of Rp volatile oil were 2.5 mg/mL against clinically isolated bacterial strains of *Pseudomonas aeruginosa* and *Acinetobacter baumanii*, 5 mg/mL against *Staphylococcus aureus*, *Escherichia coli*, *Bacillus cloacae* and *Klebsiella pneumoniae*, and 10 mg/mL against *Staphylococcus epidermidis* and *Enterococcus faecalis*. Our results, unlike previous reports [11,14], revealed that Rp volatile oil possessed a broad spectrum of antibacterial activity, possibly due to the differences in the origins of Rp used in the studies and the method used to extract the oil.

### 2.3. Components of the Rp Volatile Oil

**Figure 3:**
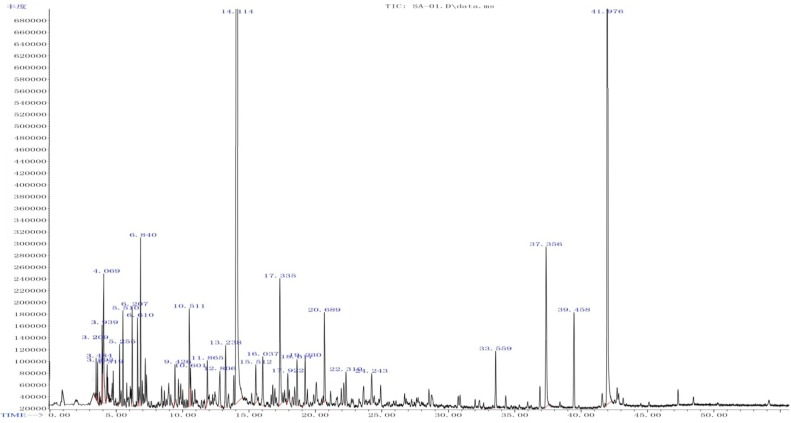
TIC-trace of Rp volatile oil.

In order to define the components of the Rp volatile oil, gas chromatography-mass spectroscopy (GC/MS) was applied. Previous phytochemical analysis based on high-performance liquid chromatography/tandem mass spectrometry (HPLC-MS/MS) revealed the presence of various phenolic compounds in Rp volatile oil, including caffeic acid conjugates, ellagic acid glycosides, and flavonol glycosides. Organic acids represent the highest content in the Rp volatile oil (66.92%), followed by formaldehyde (5.97%), alcohol-phenol (4.72%), alkanes (1.26%), ketones (1.26%), lactone (0.43%) and olefin (0.21%). The content of palmitic acid (32.67%) is the highest. In one previous study [12], only 20 components were found from the Rp volatile oil, including hexadecanoic acid (32.67%) and elaidic acid (21.20%). In our study, 29 compounds were identified using the NIST08 mass spectral database. A total ion chromatogram (TIC) trace of the volatile oil extracted from Rp leaves is shown in Figure 3. By comparing the retention times, the selected ion current (*m/z* = 51, 77, 107 and 135) profile, and the possible fragmentation mechanisms, we calculated that the structure of compound with retention time of 14.114 min was 4-hydroxy-3-methoxystyrene, which may be derived from decarboxylation of ferulic acid [15,16]. It has been reported to possess anti-inflammatory and anti-oxidative activity [17]. 4-Hydroxy-3-methoxystyrene levels increased with increasing boiling times and approximated the threshold value of 0.3 mg/L after 3 h [15].

**Figure 4:**
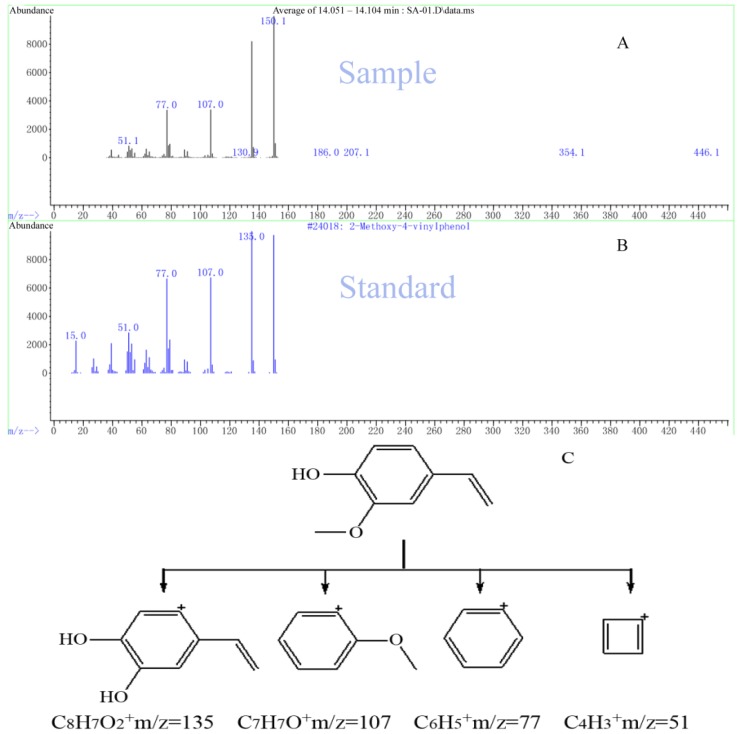
Selected ion current profile for Rp volatile oil and measured using GC/MS.(**A**) the sample with retention time of 14.114 min; (**B**) 4-hydroxy-3-methoxystyrene; (**C**) Proposed fragmentation mechanism for 4-hydroxy-3-methoxystyrene.

Table 3 summarizes the chemical composition of the Rp volatile oil. Our results showed that the alcohol-phenol constituents accounted for the highest content (81.09%) with 66.05% as 4-hydroxy-3-methoxystyrene (Table 4 and Figure 4). The yield of Rp volatile by our extraction method (0.36%, *w*/*w*) is far more better than the previous studies (<0.2%, *w/w*) [11,14]. Because of our uniform distribution experimental design, we identified more components (Table 3) that were not reported in previous reports [2,12].

**Table 3:** Chemical composition of the volatile oilextracted from *Rubus parvifolius* L. leaves.

No	RT (min)	Compound ID	Structural Formula	Formula	Content%
1	3.209	2-Acetylfuran	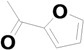	C_6_H_6_O_2_	0.28
2	3.484	Butyl formate	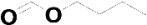	C_5_H_10_O_2_	0.46
3	3.939	5-Methyl furfural	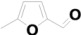	C_6_H_6_O_2_	0.55
4	4.069	Hexanoic acid	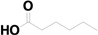	C_6_H_12_O_2_	2.03
5	4.319	*trans*-3-Hexenoic acid	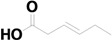	C_6_H_10_O_2_	0.32
6	5.255	Benzyl alcohol	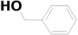	C_7_H_8_O	0.45
7	5.510	Phenyl acetaldehyde	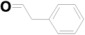	C_8_H_8_O	0.77
8	6.207	*cis*-α,α,5-Trimethyl-5-vinyl-tetrahydrofuran-2-methanol	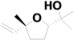	C_10_H_18_O_2_	0.88
9	6.610	α-Methyl-α-[4-methyl-3-pentenyl]oxiranemethanol	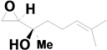	C_10_H_18_O_2_	0.78
10	6.840	Linalool	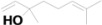	C_10_H_18_O	1.39
11	9.426	Naphthalene	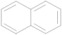	C_10_H_8_	0.37
12	10.511	2,3-Dihydrobenzofuran	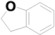	C_8_H_8_O	1.21
13	10.601	α,4-Dimethyl-3-cyclohexene-1-acetaldehyde	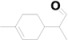	C_10_H_16_O	0.41
14	11.865	Nerol	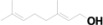	C_10_H_18_O	0.42
15	12.806	Phenylephrine	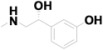	C_9_H_13_NO_2_	0.69
16	13.238	2-Methylnaphthalene	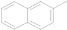	C_11_H_10_	0.81
17	14.114	4-Hydroxy-3-methoxystyrene	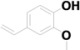	C_9_H_10_O_2_	66.05
18	15.512	1,1,6-Trimethyl-1,2-dihydronaphthalene	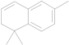	C_13_H_16_	0.57
19	16.037	Methyl 4-formylbenzoate	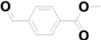	C_9_H_8_O_3_	0.77
20	17.335	4-tert-Butylbenzoic acid	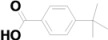	C_11_H_14_O_2_	2.22
21	17.922	Dimethylnaphthalene	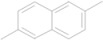	C_12_H_12_	0.37
22	18.617	α,2,6-Trimethyl-benzeneethanamine	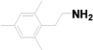	C_11_H_1__7_N	0.65
23	19.230	2-Methoxy-4-(prop-1-enyl)phenol	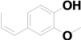	C_10_H_12_O_2_	0.64
24	20.689	Irisone	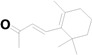	C_13_H_20_O	1.17
25	22.310	Dihydroactinidiolide	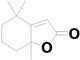	C_11_H_16_O_2_	0.52
26	24.243	2-(1,3-Butadienyl)mesitylene	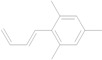	C_13_H_16_	0.52
27	33.559	Hexahydrofarnesylacetone	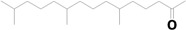	C_18_H_36_O	0.8
28	39.458	Kaur-16-ene	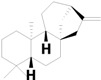	C_20_H_32_	1.36
29	41.976	3,7,11,15-Tetramethyl-2-hexadecen-1-ol	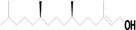	C_20_H_40_O	9.79

**Table 4:** Components of Rp volatile oil.

Compound types	Content%	Amount
Alcohol-phenol	81.09	9
Carboxylic acid	4.57	3
Ester	1.52	3
Ketone	2.25	3
Aldehyde	1.73	3
Other	5.86	8

## 3. Experimental

### 3.1. Plant Materials

*Rubus parvifolius* L. (Rp) was obtained from the Chongqing Market of Traditional Chinese Herbs and authenticated at the Chongqing Institute of Chinese Materia Medica.

### 3.2. Other Reagents

Colombia blood agar plates [18] and turbidity tubes were purchased from Chongqing Pangtong Medical Apparatus and Instrument Co., Ltd, Chongqing, China. Purified water was prepared by the Department of Pharmacy, Southwestern Hospital, Chongqing, China. Dimethyl sulfoxide (DMSO, analytical grade) was purchased from Chengdu Kelong Chemical Factory, Chengdu, China. Physiological saline was purchased from Tiansheng Pharmaceutical Group Co., Ltd., Chongqing, China.

### 3.3. Volatile Rp Oil Extraction

We extracted the volatile oil using a Clevenger apparatus in an attempt to improve the yield. A uniform distribution design was used (Table 1) to optimize extraction efficiency [11]. Rp dried leaves (200 g) were placed in a Clevenger apparatus and the powdered parts were hydro-distillated for 10 h with 2 L of purified water. Ether was added in the side arm of Clevenger apparatus to dissolve Rp volatile oil. The volatile oil was concentrated under reduced pressure to remove the ether, dehydrated by anhydrous sodium sulfate, and kept in air tight glass bottles in a refrigerator for further experiments.

### 3.4. Bacterial Strains

Three bacterial strains, *Staphylococcus aureus* (ATCC29213), *Escherichia coli* (ATCC25922) and *Pseudomonas aeruginosa* (ATCC27853) were obtained from the American Type Culture Collection (ATCC, Rockefeller, MD USA). The other bacterial strains were clinically isolated and obtained from the Department of Pharmacy, Southwestern Hospital, Chongqing, China. These strains included Gram-positive (G^+^) bacteria: *Staphylococcus aureus*, *Staphylococcus epidermidis* and *Enterococcus faecalis*; and Gram-negative (G^−^) bacteria: *Escherichia coli*, *Pseudomonas aeruginosa*, *Acinetobacter baumanii*, *Bacillus cloacae* and *Klebsiella pneumoniae* (Table 2).

### 3.5. Antibacterial Tests

To test the antibacterial effects of the volatile Rp oil, 0.048 M BaCl_2_ (1.17% w/v BaCl_2_·2H_2_O, 0.5 mL) was mixed with 0.18 M H_2_SO_4_ (1%, v/v, 99.5 mL) with constant stirring. Bacterial strains were grown on blood agar plates. Bacteria were prepared from 24 h MH broth cultures and adjusted to 0.5 McFarland turbidity equivalents. MIC of volatile oil of Rp leaves was determined by the standard CLSI (Clinical Laboratory Standards Institute) method [19]. Volatile Rp oil was weighed and dissolved in DMSO to a concentration of 10 mg/100 μL, and were then distributed at various concentrations in triplicate with a volume of 100 µL in turbidity tubes. Bacterial suspension (5 µL) with a density of 10^6^/mL in Mueller-Hinton (MH) broth was added to each tube. Positive control was a suspension of bacteria in 1 mL of MH broth, and negative control was medium without bacteria. After an incubation period of 20~24 h at 37 °C, the optical values were measured by the microdilution MH broth susceptibility assay as recommended. The lowest concentration of the test agent that prevented the appearance of turbidity was considered as the MIC. At this dilution, the test agent is bacteriostatic. Minimal bactericidal concentration (MBC) is defined as the maximal dilution of the oil that will kill a microorganism.

### 3.6. Analysis of Components

An Agilent 7890A/5975C Gas Chromatography-Mass Spectroscopy system equipped with a GC-MSD was used for analysis with ionization achieved by electron impact at 70 eV. The capillary column used was a J&W Scientific DB-1 (methylsiloxane, 30 m × 0.25 mm I.D., 0.25 µm-thick film). Experimental conditions for GC analysis of volatile Rp oil were: injection port temperature, 200 °C; interface temperature, 200 °C; column oven temperature, 80 °C for 3 min, then programmed at 3 °C/ min to 220 °C and held for 10 min; Helium (purity 99.995%) as carrier gas at a flow rate of 1.0 mL/min, and 0.20 µL injection volume. The split/splitless injector was operated in the splitless mode for 1.5 min after injection of the sample. For identification of components, the mass spectrum of each peak was recorded in the total ion current mode of the mass spectrometer within a mass range of 35 to 450 [14]. Identification of oil constituents was achieved using NIST08 mass spectral database.

### 3.7. Statistical Analysis

The data were analyzed by using uniform distribution experimental design programmed by Matlab [20]. *p* value less than 0.05 was considered statistically significant.

## 4. Conclusions

Volatile oil extracted from Rp significantly inhibits microbial growth. Rp volatile oil may represent an economic and effective antiseptic topical treatment. Taking into account that new forms of bacterial resistance to antibacterials are rising unconstrained and that the problem of pathogenic bacteria showing multi-drug resistance, our findings could be of particular useful for human and animal health. Rp volatile oil may have potential antibacterial applications. Although further studies are needed, the use of Rp volatile oil against microbial growth, especially opportunistic and pathogenic microorganisms, seems a valuable alternative to antibiotics or antibacterial compounds, especially in the case of dealing with antibiotic resistance.
